# Supplementary material for: Cognitive-behavioral statuses in depression and internet gaming disorder of adolescents: A transdiagnostic approach
Source: PLoS One. 2024 Jul 11;19(7):e0304715. doi: 10.1371/journal.pone.0304715 (PMC11239029; doi:10.1371/journal.pone.0304715)
Supplement: S1 File — (DOCX) [file pone.0304715.s002.docx]

**Supplementary materials**

**Table 1 Comparison of socio-demographic, mental disorders, and cognitive-behavioral variables between different condition groups**

| **Independent variables** | **Total (N=3147)** | **IGD group (N=97)** | **Depression group (N=1525)** | **Comorbidity group (N=368)** | **Healthy group (N=1141)** | **P-value** |
| --- | --- | --- | --- | --- | --- | --- |
|  | N (%) | N (%) | N (%) | N (%) | N (%) |  |
| ***Sociodemographic variables*** |  |  |  |  |  |  |
| Sex^a^ |  |  |  |  |  | < .001^*^ |
| Male | 1497 (48.0%) | 63 (65.6%) | 584 (38.4%) | 231 (63.1%) | 619 (54.3%) |  |
| Female | 1623 (52.0%) | 33 (34.4%) | 935 (61.6%) | 135 (36.9%) | 520 (45.7%) |  |
| Born in Hong Kong^a^ |  |  |  |  |  |  |
| Yes | 2589 (83.0%) | 71 (73.2%) | 1256 (82.6%) | 293 (79.8%) | 969 (85.4%) | .003^*^ |
| No | 530 (17.0%) | 26 (26.8%) | 265 (17.4%) | 74 (20.2%) | 165 (14.6%) |  |
| Live with parents^a^ |  |  |  |  |  |  |
| Both | 2263 (73.0%) | 75 (77.3%) | 1072 (70.9%) | 229 (63.1%) | 887 (78.4%) | < .001^*^ |
| Mother | 502 (16.2%) | 14 (14.4%) | 257 (17.0%) | 89 (24.5%) | 142 (12.6%) |  |
| Father | 166 (5.4%) | 4 (4.1%) | 90 (6.0%) | 22 (6.1%) | 50 (4.4%) |  |
| Neither | 171 (5.5%) | 4 (4.1%) | 92 (6.1%) | 23 (6.3%) | 52 (4.6%) |  |
| Mother’s educational level^a^ |  |  |  |  |  |  |
| Primary school or below | 225 (7.3%) | 11 (11.7%) | 113 (7.5%) | 32 (8.9%) | 69 (6.2%) | .079 |
| Middle school | 1393 (45.5%) | 38 (40.4%) | 713 (47.6%) | 148 (41.2%) | 494 (44.4%) |  |
| College or undergraduate | 405 (13.2%) | 16 (17.0%) | 179 (11.9%) | 44 (12.3%) | 166 (14.9%) |  |
| Master or above | 78 (2.5%) | 4 (4.3%) | 34 (2.3%) | 12 (3.3%) | 28 (2.5%) |  |
| NA (e.g., don’t know) | 963 (31.4%) | 25 (26.6%) | 459 (30.6%) | 123 (34.3%) | 356 (32.0%) |  |
| Father’s educational level^a^ |  |  |  |  |  |  |
| Primary school or below | 312 (10.1%) | 11 (11.6%) | 167 (11.1%) | 51 (14.3%) | 82 (7.3%) | < .001^*^ |
| Middle school | 1446 (47.0%) | 39 (41.1%) | 726 (48.3%) | 168 (46.2%) | 513 (46.0%) |  |
| College or undergraduate | 367 (11.9%) | 20 (21.1%) | 157 (10.5%) | 33 (9.1%) | 157 (14.1%) |  |
| Master or above | 47 (1.5%) | 2 (2.1%) | 28 (1.9%) | 3 (.8%) | 14 (1.3%) |  |
| NA (e.g., don’t know) | 905 (29.4%) | 23 (24.2%) | 424 (28.2%) | 108 (29.7%) | 350 (31.4%) |  |
|  | Mean (SD) | Mean (SD) | Mean (SD) | Mean (SD) | Mean (SD) | P-value |
| ***Sociodemographic variables*** |  |  |  |  |  |  |
| Age | 13.6 (1.3) | 13.4 (1.2) | 13.8 (1.3) | 13.6 (1.4) | 13.4 (1.3) | < .001^*^ |
| ***IGD symptoms*** | 2.3 (2.2) | 6.4 (1.5) | 1.7 (1.4) | 6.3 (1.4) | 1.3 (1.3) | < .001^*^ |
| ***Depressive symptoms*** | 19.9 (11.2) | 10.2 (3.8) | 26.7 (8.3) | 29.9 (9.2) | 9.2 (4.0) | < .001^*^ |
| ***Cognitive-behavioral variables*** |  |  |  |  |  |  |
| Self-esteem | 14.2 (3.0) | 15.3 (2.6) | 13.3 (2.6) | 12.4 (3.2) | 15.8 (2.6) | < .001^*^ |
| Dysfunctional Attitudes | 21.0 (7.3) | 19.2 (6.8) | 23.1(6.5) | 25.6 (6.9) | 16.8(6.3) | < .001^*^ |
| Hopelessness | 13.1 (3.3) | 11.9 (2.8) | 14.1 (3.1) | 15.0 (3.2) | 11.1 (2.4) | < .001^*^ |
| Coping |  |  |  |  |  |  |
| Adaptive | 16.3 (3.2) | 16.5 (3.6) | 16.5 (3.0) | 16.6 (3.3) | 16.0 (3.3) | < .001^*^ |
| Maladaptive | 12.9 (2.6) | 12.9 (2.8) | 13.4 (2.5) | 13.7 (2.6) | 12.0 (2.6) | < .001^*^ |

^*^Note: one-way ANOVA test significant comparison (0.05)

^a^The number of individuals with missing values was 27 for sex, 28 for born in HK status, 45 for Live with parents’ status, 83 for Mother’s education level, 70 for Father’s education level, 16 for identifying different condition groups.

**Table 2 Associations between sociodemographic characteristics and statuses of depression and IGD using multinomial logistic regression**

|  | **Likelihood Relative to Healthy group** | | | | | |
| --- | --- | --- | --- | --- | --- | --- |
| **Sociodemographic variable** | IGD group | | Depression group | | Comorbidity group | |
|  | ORm (95% CI) | P-value | ORm (95% CI) | P-value | ORm (95% CI) | P-value |
| Sex |  |  |  |  |  |  |
| Female | 1.00 (reference) |  | 1.00 (reference) |  | 1.00 (reference) |  |
| Male | 1.70 (1.08, 2.66) | .021^*^ | .55 (.46, .64) | < .001^*^ | 1.50 (1.17, 1.93) | .002^*^ |
| Born in Hong Kong |  |  |  |  |  |  |
| Yes | 1.00 (reference) |  | 1.00 (reference) |  | 1.00 (reference) |  |
| No | 2.27 (1.38, 3.72) | .001^*^ | 1.13 (.91, 1.41) | .268 | 1.29 (.94, 1.78) | .116 |
| Live with parents |  |  |  |  |  |  |
| Both | 1.00 (reference) |  | 1.00 (reference) |  | 1.00 (reference) |  |
| Mother | 1.09 (.58, 2.07) | .788 | 1.44 (1.14, 1.83) | .002^*^ | 2.19 (1.57, 3.03) | < .001^*^ |
| Father | .98 (.34, 2.87) | .967 | 1.57 (1.08, 2.28) | .018^*^ | 1.55 (.90, 2.69) | .116 |
| Neither | 1.07 (.37, 3.07) | .905 | 1.41 (.98, 2.02) | .066 | 1.85 (1.10, 3.12) | .021^*^ |
| Mother’s education level |  |  |  |  |  |  |
| Primary school or below | 1.00 (reference) |  | 1.00 (reference) |  | 1.00 (reference) |  |
| Middle school | .57 (.26, 1.22) | .148 | 1.00 (.71, 1.41) | .980 | .84 (.51, 1.37) | .483 |
| College or undergraduate | .58 (.23,1.49) | .259 | .84 (.56, 1.26) | .387 | .94 (.52, 1.70) | .832 |
| Master or above | .82 (1.9,3.43) | .781 | .80 (.41, 1.57) | .520 | 1.94 (.79, 4.75) | .148 |
| NA (e.g., don’t know) | .53 (.20, 1.43) | .211 | .95 (.63, 1.42) | .786 | 1.01 (.57, 1.80) | .976 |
| Father’s education level |  |  |  |  |  |  |
| Primary school or below | 1.00 (reference) |  | 1.00 (reference) |  | 1.00 (reference) |  |
| Middle school | .75 (.35, 1.62) | .462 | .69 (.51, .94) | .018^*^ | .55 (.36, .84) | .005^*^ |
| College or undergraduate | 1.21 (.49, 3.01) | .686 | .57 (.39, .84) | .004^*^ | .33 (.18, .59) | < .001^*^ |
| Master or above | 1.23 (.19, 7.89) | .828 | 1.19 (.54, 2.60) | .669 | .22 (.05, .92) | .039^*^ |
| NA (e.g., don’t know) | .70 (.26,1.86) | .468 | .61 (.42, .89) | .010^*^ | .44 (.26, .74) | .002^*^ |

Healthy group: no IGD and no depression.

ORm: multivariate odds ratio derived from the multinominal logistic regression model.

Odds ratios with p<.05 are presented with ^*^.

**Table 3. Pearson's correlations between studied variables**

| **Variables** | **1** | **2** | **3** | **4** | **5** | **6** | **7** |
| --- | --- | --- | --- | --- | --- | --- | --- |
| **1. IGD symptoms** | -- |  |  |  |  |  |  |
| **2. Depressive symptoms** | .30^*^ | -- |  |  |  |  |  |
| **3. Self-esteem** | -.22^*^ | -.59^*^ | -- |  |  |  |  |
| **4. Dysfunctional Attitudes** | .25^*^ | .57^*^ | -.44^*^ | -- |  |  |  |
| **5. Hopelessness** | .23^*^ | .64^*^ | -.56^*^ | .58^*^ | -- |  |  |
| **6. Adaptive coping** | .07^*^ | .08^*^ | .16^*^ | .05^*^ | .02 | -- |  |
| **7. Maladaptive coping** | .15^*^ | .35^*^ | -.10^*^ | .32^*^ | .26^*^ | .60^*^ | -- |

^*^*p*<0.01.

**Table 4 Associations between cognitive-behavioral characteristics and statuses of depression and IGD using multinomial logistic regression**

| **Cognitive-behavioral variables** | **Likelihood Relative to Healthy group** | | | | | | **Likelihood Relative to Comorbidity group** | | | | **Likelihood Relative to Depression group** | |
| --- | --- | --- | --- | --- | --- | --- | --- | --- | --- | --- | --- | --- |
|  | IGD group | | Depression group | | Comorbidity group | | IGD group | | Depression group | | IGD group | |
|  | ORm (95%CI) | P-value | ORm (95%CI) | P-value | ORm (95%CI) | P-value | ORm (95%CI) | P-value | ORm (95%CI) | P-value | ORm (95%CI) | P-value |
| Self-esteem | .85  (.63, 1.14) | .278 | .46  (.40, .53) | <.001^*^ | .36  (.30, .43) | <.001^*^ | 2.38  (1.73, 3.29) | <.001^*^ | 1.30  (1.12, 1.50) | .001^*^ | 1.84  (1.37, 2.48) | <.001^*^ |
| Dysfunctional attitudes | 1.32  (1.01, 1.73) | .045^*^ | 1.77  (1.56, 2.01) | <.001^*^ | 2.49  (2.06,3.01) | <.001^*^ | .53  (.39, .72) | <.001^*^ | .71  (.61, .84) | <.001^*^ | .74  (.57, .98) | .32 |
| Hopelessness | 1.25  (.92, 1.70) | .160 | 2.10  (1.82, 2.41) | <.001^*^ | 2.17  (1.79, 2.63) | <.001^*^ | .58  (.41, .80) | .001^*^ | .97  (.82, 1.13) | .670 | .60  (.44, .81) | ,001 |
| Adaptive coping | .97  (.72, 1.31) | .844 | 1.06  (.93, 1.21) | .379 | 1.10  (.92, 1.33) | .297 | .88  (.63, 1.22) | .440 | .96  (.82, 1.12) | .616 | .91  (.68, 1.24) | .562 |
| Maladaptive coping | 1.54  (1.13, 2.09) | .006^*^ | 1.48  (1.29, 1.71) | <.001^*^ | 1.47  (1.21, 1.80) | <.001^*^ | 1.04  (.75, 1.46) | .804 | 1.01  (.85, 1.19) | .927 | 1.04  (.76, 1.41) | .824 |

ORm: multivariate odds ratio derived from the multinominal logistic regression model adjusted for covariates.

Comorbidity group: both IGD and depressive symptoms; IGD group: IGD symptoms alone; Depression group: depressive symptom alone; Healthy group: neither condition.

Odds ratios with p<.05 are presented with ^*^.

**Figure 1 Prevalence of IGD group, depression group, comorbidity group and healthy group by sex**

*Note: Total sample n=3120 (27 cases with missing value on sex were excluded), Male n=1497, Female n=1623

**Table 5. Associations between cognitive-behavioral characteristics and statuses of depression and IGD using multinomial logistic regression among male participants (n=1507)**

| **Cognitive-behavioral variables** | **Likelihood Relative to Healthy group** | | | | | | **Likelihood Relative to Comorbidity group** | | | | **Likelihood Relative to Depression group** | |
| --- | --- | --- | --- | --- | --- | --- | --- | --- | --- | --- | --- | --- |
|  | IGD group | | Depression group | | Comorbidity group | | IGD group | | Depression group | | IGD group | |
|  | ORm (95%CI) | P-value | ORm (95%CI) | P-value | ORm (95%CI) | P-value | ORm (95%CI) | P-value | ORm (95%CI) | P-value | ORm (95%CI) | P-value |
| Self-esteem | 0.80  (0.61, 1.06) | 0.127 | 0.52  (0.43, 0.63) | <0.001 | 0.41  (0.33, 0.52) | <0.001 | 1.95  (1.41, 2.70) | <0.001 | 1.26  (1.03, 1.54) | 0.025 | 1.55  (3.15, 2.08) | 0.004 |
| Dysfunctional attitudes | 1.31  (1.00, 1.72) | 0.048 | 1.98  (1.64, 2.40) | <0.001 | 2.52  (1.96, 3.25) | <0.001 | 0.52  (0.37, 0.73) | <0.001 | 0.79  (0.62, 0.99) | 0.044 | 0.66  (6.49, 0.89) | 0.006 |
| Hopelessness | 1.20  (0.90, 1.61) | 0.216 | 1.96  (1.62, 2.37) | <0.001 | 1.92  (1.51, 2.44) | <0.001 | 0.63  (0.45, 0.88) | 0.007 | 1.02  (0.82, 1.27) | 0.847 | 0.61  (1.45, 0.83) | 0.002 |
| Adaptive coping | 1.23  (0.91, 1.67) | 0.175 | 1.10  (0.90, 1.36) | 0.350 | 1.16  (0.90, 1.50) | 0.257 | 1.06  (0.74, 1.52) | 0.742 | 0.95  (0.75, 1.20) | 0.667 | 1.12  (0.81, 1.54) | 0.502 |
| Maladaptive coping | 1.12  (0.82, 1.54) | 0.459 | 1.33  (1.07, 1.65) | 0.009 | 1.32  (1.00, 1.73) | 0.048 | 0.85  (0.59, 1.24) | 0.408 | 1.01  (0.78, 1.30) | 0.946 | 0.85  (0.60, 1.18) | 0.329 |

ORm: multivariate odds ratio derived from the multinominal logistic regression model adjusted for covariates.

Comorbidity group: both IGD and depressive symptoms; IGD group: IGD symptoms alone; Depression group: depressive symptom alone; Healthy group: neither condition.

**Table 6. Associations between cognitive-behavioral characteristics and statuses of depression and IGD using multinomial logistic regression among female participants (n=1629)**

| **Cognitive-behavioral variables** | **Likelihood Relative to Healthy group** | | | | | | **Likelihood Relative to Comorbidity group** | | | | **Likelihood Relative to Depression group** | | |
| --- | --- | --- | --- | --- | --- | --- | --- | --- | --- | --- | --- | --- | --- |
|  | IGD group | | Depression group | | Comorbidity group | | IGD group | | Depression group | | IGD group | | |
|  | ORm (95%CI) | P-value | ORm (95%CI) | P-value | ORm (95%CI) | P-value | ORm (95%CI) | P-value | ORm (95%CI) | P-value | ORm (95%CI) | P-value |  |
| Self-esteem | 0.89  (0.54, 1.46) | 0.652 | 0.41  (0.33, 0.50) | <0.001 | 0.33  (0.24, 0.45) | <0.001 | 2.71  (1.56, 4.70) | <0.001 | 1.24  (0.96, 1.60) | 0.103 | 2.19  (1.32, 3.62) | 0.002 |  |
| Dysfunctional attitudes | 0.99  (0.64, 1.52) | 0.965 | 1.69  (1.41, 2.04) | <0.001 | 2.98  (2.17, 4.09) | <0.001 | 0.33  (0.20, 0.55) | <0.001 | 0.57  (0.43, 0.76) | <0.001 | 0.58  (0.38, 0.91) | 0.017 |  |
| Hopelessness | 1.20  (0.72, 2.00) | 0.482 | 2.33  (1.91, 2.85) | <0.001 | 2.35  (1.71, 3.23) | <0.001 | 0.51  (0.29, 0.90) | 0.021 | 0.99  (0.75, 1.31) | 0.963 | 0.51  (0.31, 0.86) | 0.011 |  |
| Adaptive coping | 0.71  (0.45, 1.12) | 0.143 | 0.91  (0.75, 1.09) | 0.293 | 0.93  (0.70, 1.24) | 0.626 | 0.76  (0.45, 1.28) | 0.304 | 0.97  (0.76, 1.25) | 0.835 | 0.78  (0.49, 1.25) | 0.304 |  |
| Maladaptive coping | 1.85  (1.18, 2.91) | 0.007 | 1.77  (1.46, 2.14) | <0.001 | 1.61  (1.19, 2.18) | 0.002 | 1.15  (0.69, 1.93) | 0.589 | 1.10  (0.85, 1.43) | 0.478 | 1.05  (0.66, 1.66) | 0.840 |  |

ORm: multivariate odds ratio derived from the multinominal logistic regression model adjusted for covariates.

Comorbidity group: both IGD and depressive symptoms; IGD group: IGD symptoms alone; Depression group: depressive symptom alone; Healthy group: neither condition.
